# Impact of medication use on olfactory performance in older adults

**DOI:** 10.3389/fpubh.2025.1554459

**Published:** 2025-04-03

**Authors:** Maite Izco-Cubero, Fabiola Zambom-Ferraresi, Fabricio Zambom-Ferraresi, María Luisa Fernández González de la Riva, Enrique Santamaría, Joaquín Fernandez-Irigoyen, Mercedes Lachén-Montes, Juan Jose Lasarte, Maria Uzcanga-Lacabe, Secundino Fernandez, Gloria Sanjurjo San Martin, Enrique Maraví-Aznar, Nicolas Martinez-Velilla

**Affiliations:** ^1^Geriatrics and Active Aging Research Group (INGEA), Navarrabiomed, Pamplona, Navarra, Spain; ^2^Universidad Pública de Navarra (UPNA), Pamplona, Navarra, Spain; ^3^Hospital Universitario de Navarra (HUN), Pamplona, Navarra, Spain; ^4^IdiSNA, Pamplona, Navarra, Spain; ^5^CIBER of Frailty and Healthy Aging (CIBERFES), Madrid, Spain; ^6^Clinical Neuroproteomics, Navarrabiomed, Pamplona, Navarra, Spain; ^7^Universidad de Navarra (UNAV), Pamplona, Navarra, Spain; ^8^Immunology and Immunotherapy Program, Center for Applied Medical Research (CIMA), Pamplona, Navarra, Spain; ^9^Otorhinolaryngology Department, Clínica Universidad de Navarra (CUN), Pamplona, Navarra, Spain; ^10^Centro Sociosanitario Bidealdea, Cizur Menor, Navarra, Spain

**Keywords:** older people, olfactory disorders, medication, olfaction, polypharmacy, olfactory dysfunction, ageing

## Abstract

**Introduction:**

Olfactory dysfunction impacts quality of life, safety, and nutrition. Despite its relevance among older adults, the role of medications in influencing olfactory performance remains understudied. This research investigates whether olfactory alterations in older adults are associated with the type or number of medications prescribed.

**Methods:**

An observational cross-sectional study was conducted with 107 participants (mean age of 86.1 ± 5.1 years). Olfactory performance was evaluated using the Sniffin’ Sticks Test (SST). Functional capacity, cognitive function and the number and type of medications were also assessed.

**Results:**

The analysis demonstrated a correlation between better olfactory performance and higher cognitive function. An inverse correlation was found between the age of participants and olfactory identification. While polypharmacy (intake of five or more medications) did not show a significant association with olfactory dysfunction, the intake of laxatives was associated with poorer olfactory threshold performance (−1.21, 95% CI −2.07 to −0.34; *p* = 0.008). In contrast, proton pump inhibitors (PPIs) (1.14, 95% CI 0.07 to 2.21; *p* = 0.04) and vitamin D (1.09, 95% CI 0.03 to 2.15; *p* = 0.04) intake were linked to improved olfactory identification.

**Discussion:**

These findings suggest that certainmedications influence olfactory performance; however, further research is needed to clarify the effects of different drug classes on olfaction.

## Introduction

Olfaction is the process by which odors are detected through sensory impulses transmitted via olfactory neurons (1, 2). It is assessed based on three key parameters: threshold detection, identification, and discrimination. A sense of smell is essential for numerous processes, including eating habits, detection of environmental dangers, and social communication (3). It can be assessed through a range of olfactory tests and is typically classified as normosmia (normal olfaction), hyposmia (reduced olfaction), and anosmia (absence of olfaction) (1).

The prevalence and severity of olfactory dysfunction increase significantly with age, leading to a decline in quality of life. The reduction in the ability to recognize and differentiate between odors has been associated with a diminution of physical health, daily safety, and food satisfaction, as well as increased depressive symptoms and mortality rates (2, 4–9). Olfactory impairment is highly prevalent among older adults, with a prevalence of 13.9% in individuals aged 65. The frequency of this impairment increases significantly with age, affecting 50% of those between 65 and 80 years and reaching 80% in individuals over the age of 80 (4). However, fewer than 25% of patients with olfactory dysfunction are aware of this impairment (10).

Despite the significance of olfaction in human behavior and the high prevalence of dysfunction in older people, olfactory impairment has been disregarded and infrequently evaluated in clinical settings (11). Several factors contribute to age-related decline in olfactory function, including anatomical and physiological changes, surgical interventions, medications, trauma, environmental conditions, and diseases (12, 13). Olfactory dysfunction is an early symptom of several neurodegenerative diseases, often appearing decades before motor or cognitive decline (10, 14). It serves as a clinical marker for prodromal stages of conditions such as Alzheimer’s and Parkinson’s disease; however, the underlying cellular and molecular mechanisms remain poorly understood (1, 11, 13, 15).

Medication use is a recognized factor influencing olfactory dysfunction. Older patients, who make up 13–16% of the population, receive 40% of the prescribed drugs, primarily due to multimorbidity. Additional factors that increase drug use include the availability of medications, pharmacological guidelines recommendations, and the exacerbation of illnesses due to poor adherence to complex treatments (16–18). Medications have the potential to affect the physiological chemosensory processes involved in olfactory perception by changing the chemical or ionic environment, modifying the functioning of receptors, or altering neurotransmission (19).

Relatively few studies have assessed the influence of different types of medications on olfaction, as standard guidelines for drug development do not require testing the impact of medication on smell. Nevertheless, more than 70 drugs with affecting olfaction have been identified in clinical trials across all main pharmacological categories (12, 19–21). Moreover, it is noteworthy to highlight that older adults are frequently excluded from clinical trials, resulting in a dearth of data on the efficacy and safety of numerous drugs (18). Ottaviano et al. (22) conducted a study addressing the influence of number and types of medications on olfaction in the older adults with a mean age of 74 years. A correlation between a worse sense of smell at the olfactory threshold and the use of calcium channel blockers, *β*-blockers, and acetylsalicylic acid was found. In addition, a worse sense of smell during olfactory identification among those consuming acetylsalicylic acid and potassium-sparing diuretics was observed (22). Further studies are required to validate these preliminary results. In this context, this study aimed to investigate whether olfactory dysfunction in older adults is related to the type or number of medications prescribed.

## Materials and methods

### Study design and participants

This observational cross-sectional study was conducted from March 2021 to February 2023, following the “Olfactory Characterization and Training in Older Adults: Protocol Study” the protocol, published in November 2021 (23). Olfactory capacity was assessed using the Sniffin’ Sticks Test (SST), a psychophysical tool providing a semi-objective measure of olfactory performance. The SST extended version ranges from 1 to 48 points, and comprises three subtests: threshold, discrimination, and identification (24). A score of 31 points or higher indicates normosmia, a score between 17 and 30 points implies hyposmia (impaired olfactory function), and 16 or lower suggests functional anosmia (25, 26). Age, height, weight, and body mass index (BMI) were also recorded, as well as the quantity, dose, active principle, and type of drugs prescribed to the participants.

Functional capacity was assessed using the Short Physical Performance Battery (SPPB), which evaluated leg strength, gait speed, and balance on a 12-point scale (27). Disability was measured using the Barthel Index of Independence in Activities of Daily Living (28). For the cognitive evaluation, the Mini-Mental State Examination (MMSE) score and Symbol Digit Modalities Test (SDMT) were used Cognitive function was assessed using the Mini-Mental State Examination and the Symbol Digit Modalities Test. The MMSE, a widely used screening tool, evaluates orientation, memory, attention, language, and visuospatial abilities, with a maximum score of 30 (29, 30). The SDMT assesses cerebral dysfunction and information processing speed. The test’s maximum score of 110 was determined by the number of correct symbol/digit substitutions within the allotted time (31). Perceived health was quantified using the Visual Analog Scale (VAS) from the EuroQol 5-Dimension 3-Level (EQ-5D-3L) questionnaire, where 0 represents the worst imaginable health state and 100 representing the best imaginable health state (32).

Recruitment was conducted in three different locations: the acute geriatric unit of the Hospital Universitario de Navarra (GU), Geriatric Outpatient Clinic at the Hospital Universitario de Navarra (GC) and nursing homes (NH). A total of 107 participants were recruited using a convenience sampling method. The study adhered to the principles of the Declaration of Helsinki (54) and was approved by the Hospital Universitario de Navarra Clinical Research Ethics Committee in October 2020 (PI_2020/113). All the participants or their legal representatives signed an informed consent form.

The inclusion criteria were: (i) age over 65 years; (ii) an MMSE score of at least 21 (or 23 for participants with higher educational attainment); and (iii) clinical stability. Exclusion criteria included a diagnosis of any neurodegenerative disease or nasal sinus pathology (e.g., sinusitis, previous nasal surgery, nasal polyps, or nasal congestion at the time of testing). Other exclusion criteria were a recent upper respiratory tract infection (within 2 weeks), use of medications known to impair olfactory performance, or a history of COVID-19 with associated olfactory dysfunction.

### Outcomes

The primary outcome was to investigate whether olfactory dysfunction in older adults is related to the type or number of medications prescribed. This was measured using the SST, which assesses olfactory performance across three subtests: Threshold, Discrimination, and Identification (TDI score).

The secondary outcomes included evaluating the impact of polypharmacy (use of five or more drugs) on olfactory function, and assessing the relationship between olfactory performance and cognitive function, functional capacity, and quality of life through different tests: MMSE, SPPB, Barthel Index, SDMT, and VAS from EQ-5D-3L for health perception.

### Statistical analysis

Statistical analyses were performed using R programming language, version 4.3.3. (R Foundation, Vienna, Austria). Descriptive analysis was performed by calculating the means and standard deviations of the evaluated variables. Pearson’s correlation analysis was performed to assess the relationships between olfactory performance and functional capacity, cognitive evaluation, and other clinical variables.

Three multivariate regression models were developed in this study. The first included medications that presented a decrease in the TDI score, the second model comprehend medications showing a decrease in olfactory threshold, and the third and last, included medications increasing olfactory identification. Regression analysis of all subsets was used to choose the model variables. Multicollinearity of regression models was evaluated calculating variance inflation factors (VIFs) for all independent variables included in the analyses. This technique finds the optimum model based on the Akaike information criterion (AIC).

Subsequently, the participants were divided into two different subgroups: participants without polypharmacy (taking fewer than five drugs) and those with polypharmacy (taking five or more drugs), and participants using a type of drug versus no use. Student’s *t-*test for independent samples was used to compare the groups. Statistical significance was set at *p* < 0.05.

## Results

The study included 107 participants, of whom 58 (54.2%) were women, with a mean age of 86.1 ± 5.1 years. Participants were recruited from three sites: 63 from the Acute Geriatric Unit (GU), 30 from the Geriatric Outpatient Clinic (GC), and 14 from nursing homes (NH). The study population demonstrated independence in performing basic daily activities (Barthel Index mean: 83.40), exhibited frailty (SPPB mean: 6.74), and had no diagnosed cognitive impairment (MMSE mean: 26.68). The mean TDI score was 17.8 ± 5.8, with subtest scores of 2.8 ± 2.4 for threshold, 7.5 ± 2.3 for discrimination, and 7.5 ± 2.7 for identification. According to the SST reference values, 50.5% of participants were classified as having hyposmia, 48.5% as having functional anosmia, and 1% as having normosmia. Additionally, 83 participants reported polypharmacy, taking five or more drugs, with an average of 8.5 ± 3.8 medications per participant (Table 1).

**Table 1:** Demographic, olfactory performance test and clinical data of participants.

Variable	Mean (SD)	Range
Demographic data		
Age, yr	86.06 (5.06)	69–99
Weight, kg	66.24 (13.65)	38.5–112
Height, m	1.58 (0.87)	1.36–1.82
BMI	26.59 (4.51)	16.49–39.21
Olfactory performance test data		
Sniffin’ Sticks Test	17.76 (5.47)	6–32.5
Olfactory threshold	2.82 (2.37)	1–10
Olfactory discrimination	7.54 (2.33)	2–14
Olfactory identification	7.47 (2.69)	1–13
Clinical data		
MMSE	26.68 (2.41)	21–30
SPPB scale	6.74 (3.31)	0–12
Barthel Index	83.40 (17.21)	25–100
SDMT	13.35 (8.78)	0–34
VAS from EQ-5D-3L	65.20 (19.81)	5–100
Number of drugs taken	8.49 (3.75)	0–18

BMI, Body Mass Index; MMSE, Mini-Mental State Examination; SPPB, Short Physical Performance Battery; SDMT, Symbol Digit Modalities Test; VAS, Visual Analog Scale; EQ-5D-3L, EuroQol5-Dimension3-Level questionnaire.

A positive correlation was observed between higher SST scores and MMSE (*r*^2^ = 0.03, *p* = 0.04) and SDMT (*r*^2^ = 0.09, *p* = 0.005) scores. Additionally, an inverse correlation was found between participant age and olfactory identification (*r*^2^ = 0.05, *p* = 0.01). No additional correlation was observed between the TDI score and the other variables analyzed, including the number of drugs taken by the participants (*r*^2^ = −0.003, *p* = 0.39).

**Figure 1:**
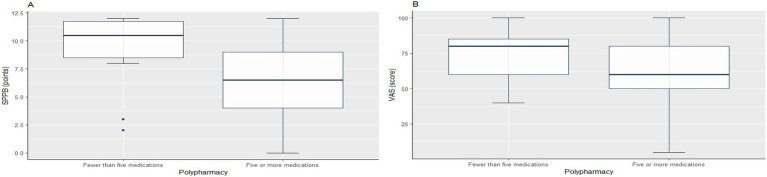
Differences in Short Physical Performance Battery (SPPB) and Visual Analog Scale (VAS) scores in polypharmacy and nonpolypharmacy groups. **(A)** Boxplot of SPPB scores between non-polypharmacy (taking fewer than five medications) and polypharmacy (taking five or more medications) groups. **(B)** Boxplot of VAS scores between non-polypharmacy (taking fewer than five medications) and polypharmacy (taking five or more medications) groups.

To assess the impact of polypharmacy on functional capacity, participants were stratified into two subgroups: those taking fewer than five medications and those taking five or more. Participants in the polypharmacy group exhibited significantly lower SPPB scores (−2.7, 95% CI −5.4 to −0.005; *p* = 0.04; Cohen’s d = 0.83) and VAS scores (−14.1, 95% CI −23.8 to −4.4; *p* = 0.006; Cohen’s d = 0.43) (Figure 1). No significant differences were observed between TDI scores and polypharmacy status (*p* = 0.53).

**Figure 2:**
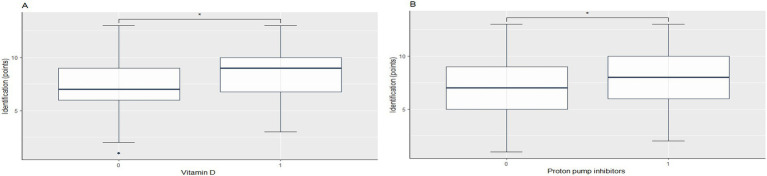
Influence of Vitamin D and Proton Pump Inhibitors on Olfactory Identification Performance. **(A)** Identification score in participants not taking vitamin D (0) versus those taking vitamin D (1). **(B)** Identification score in participants not taking proton pump inhibitors (0) versus those taking proton pump inhibitors (1).

Analysis of olfactory function in relation to medication type identified a statistically significant association between poorer olfactory threshold performance and laxative use (−1.21, 95% CI −2.07 to −0.34; *p* = 0.008; Cohen’s d = 0.51). In contrast, the results showed a statistically significant difference between better olfactory identification scores and intake of proton pump inhibitors (PPIs) (1.14, 95% CI 0.07 to 2.21; *p* = 0.04; Cohen’s d = −0.43) and vitamin D (1.09, 95% CI 0.03 to 2.15; *p* = 0.04; Cohen’s d = −0.41) (Figure 2, Table 2).

**Table 2:** Type of medication taken by participants, olfactory performance alteration in Sniffin’ sticks test, between-group difference (95% CI) and *t*-test *p*-value.

Type of medication / Olfactory performance alteration	No taking medication	Taking medication
Laxatives / Threshold		
*n*	88	19
Mean (SD)	3.02 (2.49)	1.81 (1.32)
Between-group difference (95% CI)	−1.21 (−2.07 to −0.34)	
*p*-value between groups	0.008	
Proton pump inhibitors / Identification		
*n*	44	63
Mean (SD)	6.78 (2.71)	7.92 (2.60)
Between-Group Difference (95% CI)	1.14 (0.07 to 2.21)	
*p*-value between groups	0.04	
Vitamin D / Identification		
*n*	75	32
Mean (SD)	7.13 (2.77)	8.22 (2.37)
Between-group difference (95% CI)	1.09 (0.03 to 2.15)	
*p*-value between groups	0.04	

The use of angiotensin receptor/neprilysin inhibitors (ARNIs), cholinesterase inhibitors, iron, potassium-sparing diuretics, prokinetic agents, prostaglandin analogs, and serotonin-norepinephrine reuptake inhibitors (SNRIs) was associated with lower olfactory threshold scores. However, these findings were not statistically significant due to the limited sample size.

None of the multivariate analyses showed any statistically significant differences, and the variables in all three different analyses lost statistical significance. However, no VIFs exceeded the value of five, indicating that collinearity was not significant in the models.

## Discussion

This study investigated whether olfactory function in older adults is associated with the type or number of medications prescribed. The findings indicate a high prevalence of olfactory dysfunction among older adults, with 50.5% of participants classified as having hyposmia and 48.5% as having functional anosmia. These results support other studies that point out the frequent prevalence of olfactory impairment in this age group (4–10).

Our data suggest a significant association between olfactory performance and cognitive function, as indicated by the positive correlation between TDI scores and MMSE and SDMT results. This is consistent with studies that have linked olfactory function and cognition (33–37). Research suggests that cognitively normal individuals with lower odor identification scores are at increased risk of developing mild cognitive impairment (MCI). Furthermore, individuals with MCI are more likely to experience cognitive decline and progress to Alzheimer’s disease compared to those without olfactory dysfunction (34–36). Moreover, it has been observed that if the test used combines odor identification and odor threshold, the prediction rate of cognitive dysfunction increases (37).

An inverse correlation was observed between age and olfactory identification, whereas no significant correlation was found with olfactory threshold or discrimination. This may be explained by the fact that odor identification involves higher-order cognitive processing, including memory recall and recognition of previously encountered scents (34, 38). No other associations were found between olfactory function and weight, height, BMI, SPPB, Barthel Index, VAS or the number of drugs taken by participants.

Unexpectedly, no significant correlation was found between olfactory function and the number of medications taken. This contrasts with previous studies suggesting that polypharmacy may exacerbate olfactory impairment (22). Otherwise, there was a statistically significant difference between the subgroups taking five or more drugs and lower SPPB and VAS scores. These results are consistent with previous studies associating polypharmacy with a lower SPPB score (39) and worse self-rated health (40).

Our findings also indicate that specific medications may influence olfactory performance. Laxative use was associated with poorer olfactory threshold scores, though no established mechanism directly linking laxatives to olfactory decline has been identified. The study also identified an association between the use of ARNIs, cholinesterase inhibitors, iron, potassium-sparing diuretics, prokinetic agents, prostaglandin analogs or SNRIs with poorer olfactory threshold performance. Nevertheless, these findings were not statistically significant due to the limited sample size and therefore have limited clinical significance. Currently, no study has directly connected ARNIs to olfactory dysfunction. However, growing evidence suggests that olfactory impairment and cardiovascular health are related (41, 42), and various cardiovascular medications have been reported to affect olfactory function (41, 43). Several studies have explored the link between iron and olfactory dysfunction and have found that both high and low iron levels can impair the sense of smell. This emphasizes the importance of maintaining balanced iron levels for olfactory health (44, 45). Although there is no information connecting prokinetic medications and olfactory impairment, some studies have provided indirect evidence that could suggest a possible relationship. Prokinetic agents can cross the blood–brain barrier, affecting the central nervous system and causing neurological side effects. Olfactory abnormalities could possibly be a side effect of these drugs because of the strong connection between the olfactory system and the central nervous system (46, 47). There was no established connection between the use of potassium-sparing diuretics, SNRIs, prostaglandin analogs or cholinesterase inhibitors and olfactory performance. In a study conducted by Ottaviano et al. (22), worsening of olfactory identification and intake of potassium-sparing diuretics were observed. The literature does not list olfactory dysfunction as a known side effect of any of these medications; however, these findings allow us to expand this area of knowledge.

The use of PPIs and vitamin D was associated with improved olfactory identification scores. PPI, due to its effect on gastric secretion, is one of the most widely used drugs to treat gastroesophageal reflux disorders. However, this pathology also manifests as exacerbation of extraesophageal symptoms (48, 49). PPIs may potentially improve the nasal mucosa environment while reducing gastric acidity, and hence be the cause of the positive influence in olfaction (49, 50). Vitamin D acts as a neurosteroid hormone in the brain, spinal cord, and olfactory system proving the plausible relationship between the nervous system and vitamin D deficiency (51, 52). Moreover, there is scientific evidence that relates olfactory dysfunction with vitamin D insufficiency (52), and the improvement of olfaction after vitamin D intake (53).

A key limitation of this study is the small sample size, which may have affected the statistical power of the analysis. With only 107 participants, the study might not have been sufficiently powered to detect associations in the multivariate models. Another critical factor to consider is the potential influence of confounding variables, which may have affected the observed associations between medication use and olfactory function. While adjustments were made for age, cognitive performance, and functional capacity, other unmeasured factors such as comorbidities, lifestyle factors, and dietary habits could have influenced the results. Future studies should incorporate a larger cohort that also account comorbidities, lifestyle habits, and dietary influences, to better elucidate the mechanisms contributing to olfactory impairment in this population. Despite these limitations, the study provides valuable insights into the complex relationship between medication intake and olfactory function in older adults.

One of the strengths of this study is that it addresses this relevant impairment in older populations. Given the high mean age of participants (86.1 ± 5.1 years), the research offers valuable insights into a demographic often excluded from clinical trials. Only few studies have analyzed the relationship between olfactory dysfunction in this age group and the type or number of medications prescribed. However, with a sample size of 107 participants, the research might not have enough power to identify smaller effects or generalize findings to larger populations. In addition, the study has limited control over medication regimens. 77.6% of participants reported polypharmacy, taking five or more drugs, with an average of 8.5 ± 3.8 medications per participant. Therefore, the complexity of drug interactions and individual health conditions might mask the specific effects of each medication on olfaction.

These results emphasize the potential impact of the type of medication prescribed on olfactory dysfunction in older adults. In clinical practice, olfactory impairment is frequently overlooked, despite the fact that it can have a significant impact on safety, nutritional intake, and quality of life. Clinicians should integrate regular olfactory assessments into geriatric care to identify and address olfactory dysfunction. Simple screening tools, such as the SST could aid in the early identification of olfactory deficits, allowing for timely interventions. Additionally, medication reviews should take into account not only polypharmacy but also the potential impact of specific drug classes on olfactory function. In this study, laxative use was associated with poorer olfactory thresholds, while PPIs and vitamin D intake were linked to better olfactory identification. Although these findings require further validation, clinicians should remain aware of the potential sensory side effects of medications. Given the impact on nutritional intake and safety, healthcare providers should monitor dietary habits in affected patients and provide nutritional counseling or supplementation when necessary. Integrating olfactory assessments into routine clinical evaluations and revising medication management could help mitigate this impairment, improving the quality of life and well-being of older adults.

## Conclusion

This study highlights the intricate nature of the relationship between medication intake and olfactory function among older adults. It is crucial to emphasize that a limited number of studies have investigated the impact of different types of medication on olfaction, and clinical trials frequently exclude older adults from their participant pool. Our findings revealed that, while polypharmacy did not exhibit a significant association with overall olfactory dysfunction, some medications had a discernible effect on olfactory performance. Nevertheless, the pharmacological profiles of the participant were complex, which may have led to masking effects that altered the outcomes. Further research is necessary to comprehend the specific effects of each medication on the olfactory system and the role of polypharmacy in olfactory dysfunction. Gaining insights into this relationship could facilitate the development of interventions aimed at preserving or restoring olfaction in older adults, ultimately improving their overall health and quality of life.

## Data Availability

The raw data supporting the conclusions of this article will be made available by the authors, upon reasonable request.
